# Medicinal Plants in the Prevention and Treatment of Chronic Diseases

**DOI:** 10.1155/2012/458274

**Published:** 2012-09-18

**Authors:** Mohamed Eddouks, Debprasad Chattopadhyay, Vincenzo De Feo, William C. Cho

**Affiliations:** ^1^Moulay Ismail Unversity, BP 21, Errachidia 52000, Morocco; ^2^ICMR Virus Unit, Division of Ethnomedicine, ID & BG Hospital, General Block 4, 57 Dr. Suresh C Banerjee Road, Kolkata 700 010, India; ^3^Dipartimento di Scienze Farmaceutiche e Biomediche, Università degli Studi di Salerno, Via Ponte don Melillo, 84084 Fisciano, Italy; ^4^Department of Clinical Oncology, Queen Elizabeth Hospital, Kowloon, Hong Kong

Since the dawn of human civilization, human beings have found remedies within their habitat and have adopted different therapeutic strategies depending upon climatic, phytogeographic, sociocultural, floral, and faunal characteristics. Traditional systems thus contain beliefs and practices in order to avoid, prevent, or avert ailments, which constitute traditional preventive medicine. The use of medicinal herbs and herbal medicine is an age-old tradition and the recent progress in modern therapeutics has stimulated the use of natural product worldwide for diverse ailments and diseases. The educated public and health care professionals have enormous interests in the medicinal uses of herbs, but there is a great deal of confusion about their identification, effectiveness, therapeutic dosage, toxicity, standardization, and regulation. According to WHO, traditional medicine is popular in all regions of the world and its use is rapidly expanding even in developed countries. For example, in China, traditional herbal preparations account for 30–50% of the total medicinal consumption and now the annual global market for herbal medicine is over 60 billion USD. Thus, Western trained physicians should not ignore the impact of traditional medicine on their patients.

This special issue on medicinal plants in the prevention and treatment of chronic diseases is an attempt to summarize the current knowledge of promising traditional medicines and their phytophores to compounds tested against diverse chronic diseases. The therapeutic properties and structure activity relationship of some important and potentially useful phytoformulations are addressed with a focus on how these age-old wisdom can led to the development of useful therapeutics lead for preclinical or clinical evaluation. Manuscripts in this special issue covered several aspects of recent developments in the fields of (1) natural substances as lead compounds in chronic and degenerative diseases research, (2) natural products involved in the prevention of chronic diseases, (3) herbal pharmacotherapy and phytochemical studies, (4) role of functional foods and nutraceuticals in chronic diseases, and (5) studies involving toxicology and pharmacological and toxicological mechanisms of action of medicinal plants used in the treatment and prevention of chronic diseases. In-depth information prepared by experts from diverse fields provide the use of diverse medicinal herbs and their active components as antioxidants, antidiabetic, antihypertensive, antiatherosclerosis, gastroprotective, analgesic, anticancer, antidepressant, antiasthma, antiobesity, antiatherosclerosis, antimicrobial, anti-inflammatory agents and as immunomodulators, along with their safety issues and toxic effects.

In the coming days, more issues of eCAM will be released to offer researchers working on diverse aspects of medicinal plants with a complete coverage of ethnology, pharmacology, toxicology, and medicinal properties. This special issue will provide essential materials to those who are working in the fields of traditional systems of medicine and drug industry. It is the outcome of our research involvement for the last two decades with the subject and consultations among biomedical scientists and clinicians. Our group of four coeditors active in phytotherapy research in three continents has been very pleased to receive a substantial feedback of 59 submissions to this special issue. 

